# Efficacy and safety of lenvatinib plus gefitinib in lenvatinib-resistant hepatocellular carcinomas: a prospective, single-arm exploratory trial

**DOI:** 10.1038/s41392-024-02085-8

**Published:** 2024-12-09

**Authors:** Yaoping Shi, Dan Cui, Lei Xia, Donghua Shi, Guangxin Jin, Siying Wang, Yan Lin, Xiaoyin Tang, Jiachang Chi, Tao Wang, Meng Li, Zicheng Lv, Jiaojiao Zheng, Qi Jia, Wu Yang, Zhen Sun, Fan Yang, Hao Feng, Shengxian Yuan, Weiping Zhou, Wenxin Qin, Rene Bernards, Haojie Jin, Bo Zhai

**Affiliations:** 1 Department of Interventional Oncology, State Key Laboratory of Systems Medicine for Cancer, Renji Hospital, Shanghai Jiao Tong University School of Medicine, Shanghai, PR China; 2 Department of Liver Surgery, State Key Laboratory of Systems Medicine for Cancer, Renji Hospital, Shanghai Jiao Tong University School of Medicine, Shanghai, PR China; 3 Shanghai Cancer Institute, State Key Laboratory of Systems Medicine for Cancer, Renji Hospital, Shanghai Jiao Tong University School of Medicine, Shanghai, PR China; 4 Department of Pharmacy, Renji Hospital, Shanghai Jiao Tong University, Shanghai, PR China; 5 The Third Department of Hepatic Surgery, Eastern Hepatobiliary Surgery Hospital, Third Affiliated Hospital, Naval Medical University, Shanghai, PR China; 6 Division of Molecular Carcinogenesis, Oncode Institute, The Netherlands Cancer Institute, Amsterdam, The Netherlands

**Keywords:** Drug development, Target identification

## Abstract

Lenvatinib, a multi-kinase inhibitor, has been approved as first-line treatment for advanced hepatocellular carcinoma (HCC), but its efficacy is limited. We have shown previously that lenvatinib and epidermal growth factor receptor tyrosine kinase inhibitor (EGFR-TKI) combination therapy overcomes lenvatinib resistance in HCC with high level of EGFR expression (EGFR^high^). We present here the results of a single-arm, open-label, exploratory study of lenvatinib plus the EGFR-TKI gefitinib for patients with HCC resistance to lenvatinib (NCT04642547; *n* = 30). Only patients with EGFR^high^ HCC and progressive disease after lenvatinib treatment were recruited in the study. The most frequent adverse events of all grades were fatigue (27 patients; 90%), followed by rash (25 patients; 83.3%), diarrhea (24 patients; 80%), and anorexia (12 patients; 40%). Among 30 patients, 9 (30%) achieved a confirmed partial response and 14 (46.7%) had stable disease according to mRECIST criteria. Based on RECIST1.1, 5 (16.7%) achieved a confirmed partial response and 18 (60%) had stable disease. The estimated median progression free survival (PFS) and overall survival (OS) time were 4.4 months (95% CI: 2.5 to 5.9) and13.7 months (95% CI: 9.0 to NA), respectively. The objective response rate (ORR) of the patients in the present study compares very favorable to that seen for the two approved second line treatments for HCC (cabozantinib ORR of 4%; regorafenib ORR of 11%). Given that this combination was well-tolerated, a further clinical study of this combination is warranted.

## Introduction

Liver cancer is the third leading cause of cancer-related death and ranks sixth in terms of incidence worldwide.^1^ With the increase in non-alcoholic fatty liver disease (NAFLD) and obesity, liver cancer is increasing in Europe and the United States.^2–4^ Hepatocellular carcinoma (HCC) accounts for about 85%–90% of all primary liver malignancies. Due to the inconspicuous symptoms and rapid progression of HCC, most patients are diagnosed at an advanced stage, making systemic treatment especially important in the clinic.^5^ Unfortunately, the most prevalent oncogenic mutations are currently undruggable, such as mutations of *TP53*, *CTNNB1* and the *TERT* promoter, and few molecular targeted therapies have proven to be effective.^6,7^ HCC is a typical solid tumor with abundant blood vessels. The abnormal vascular network and angiogenesis contribute to its growth and progress. Therefore, in both first-line and second-line settings, effective treatments for advanced HCC often include anti-angiogenic drugs targeting vascular endothelial growth factor receptor (VEGFR) and platelet-derived growth factor receptor (PDGFR).^8^

Lenvatinib is an oral multi-kinase inhibitor that inhibits VEGFR 1-3, fibroblast growth factor receptors (FGFR1-4), PDGFR α, KIT, and RET. Lenvatinib was found to be non-inferior to sorafenib for the primary endpoint of overall survival (OS)^9^ and was approved for the treatment of unresectable HCC as a standalone first-line therapy in 2018. However, the objective response rate (ORR) for lenvatinib is only 24.1%, albeit that this is higher than that of sorafenib (ORR: 9.2%).^9^ A number of clinical trials are being or have been conducted to assess the combination of lenvatinib with other standard treatments, such as transarterial chemoinfusion (NCT04053985), Transarterial chemoembolization (TACE) (NCT03905967, NCT05220020), systemic chemotherapy (NCT04170179), or PD-1 antibody (NCT03713593, NCT04443309). However, most trials lack a rational molecular mechanism to justify the combination, which limits chances of success.^10^

In our previous study, we used kinome-centered CRISPR-Cas9 library for synthetic lethal screening to determine the genes that drive lenvatinib resistance. We found that targeting EGFR gave EGFR^high^ HCC cells a response to levatinib in vitro and in vivo, but EGFR^low^ HCC cells did not respond. We found that targeting EGFR confers responsiveness of EGFR^high^ HCC cells to lenvatinib both in vitro and in vivo, as EGFR^low^ HCC cell lines did not respond. Furthermore, the EGFR/PAK2/ERK5 axis was identified to mediate the drug resistance of lenvatinib by a series of biological and biochemical studies. Also, we carried out an exploratory clinical trial (trial identifier NCT04642547) to test the antitumor efficacy and safety of lenvatinib plus gefitinib in HCC patients who were unresponsive to lenvatinib treatment. The 12 patients who were recruited in the early stage showed meaningful, but prlininary clinical responses.^11^ Several other pre-clinical studies also suggested that EGFR-associated signaling can trigger lenvatinib-resistance.^12–17^ Together, these findings provide a clear rationale to test lenvatinib plus EGFR inhibitor as a potential combination therapy approach for HCC in the clinic.

Herein, we present the final safety and antitumor activity results of the investigator-initiated, first-in-human, and exploratory clinical study, which tests the combination of lenvatinib plus EGFR inhibitor gefitinib in 30 EGFR^high^ HCC patients, who expressed high protein level of EGFR and showed disease progression upon lenvatinib treatment.

## Results

### Patient characteristics

**Fig. 1:**
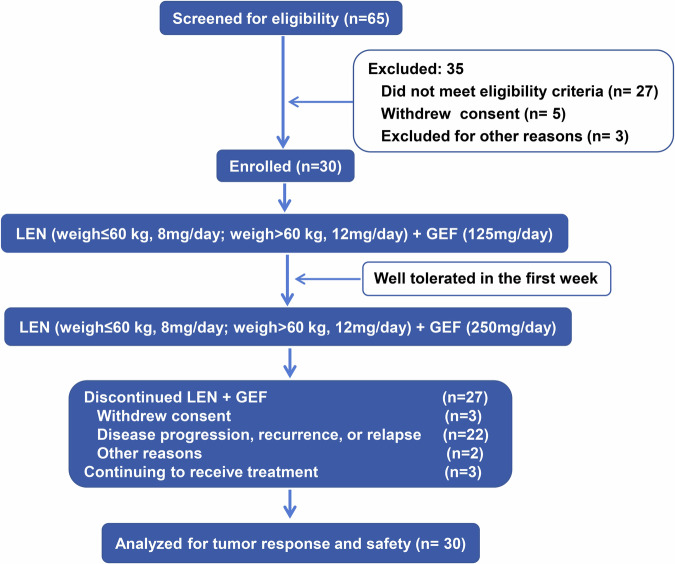
Flowchart of the study. AE, adverse event; LEN, lenvatinib; GEF, gefitinib

We initially screened 65 patients between 2020 and 2023 at Renji hospital in China and ultimately 30 patients were enrolled and analyzed for safety and therapy response (Fig. 1). Table 1 shows their baseline demographics. All patients had high expression level of EGFR in the tumor biopsies^11^ and showed disease progression upon lenvatinib therapy before enrollment. Fourteen patients (46.7%) underwent anti-PD-(L)1 immunotherapy prior to enrollment and ten patients (33.3%) received two or more targeted drugs prior to enrollment. Among them, seven patients (23.3%) received anti-PD-(L)1 therapy and other targeted therapies in addition to lenvatinib therapy. Other treatments include transarterial chemoembolization (TACE) and/or hepatic artery infusion chemotherapy (HAIC) (73.3%), microwave ablation (MWA)/radiofrequency ablation (RFA) (70%), surgery (66.7%) and radiotherapy (26.7%).

**Table 1:** Patient baseline demographics

Patient characteristic	
Age, years	
<60, No.(%)	19 (63.3)
≥60, No.(%)	11 (36.7)
Gender, No. (%)	
Male	25 (83.3)
Female	5 (16.7)
Bodyweight, Kg, No. (%)	
<60	17 (56.7)
≥60	13 (43.3)
Etiology, No. (%)	
Hepatitis B	27 (90)
Hepatitis C	0
Others	3 (10)
Liver cirrhosis, No. (%)	21 (70)
ECOG-PS score, No. (%)	
0	5 (16.7)
1	25 (83.3)
**Child-Pugh class, No. (%)**	
A	24 (80)
B	6 (20)
WBC, ×10^9^/L, median (SD)	5.7 (3.8)
RBC, ×10^12^/L, median (SD)	4.5 (0.8)
PLT, ×10^9^/L, median (SD)	148.2 (77.8)
ALT, U/L, median (SD)	33 (22.1)
AST, U/L, median (SD)	44 (31)
ALB, g/L, median (SD)	40 (4.3)
TBil, umol/L, median(SD)	16.9 (11.2)
PT, seconds, median (SD)	11.8 (0.9)
AFP, ng/ml, median (SD)	386.5 (11347.8)
<400,No.(%)	15 (30)
≥400,No.(%)	15 (30)
Intrahepatic tumors, No. (%)	
Single	0 (0)
Multiple	30 (100)
Main tumor size	
<5, No. (%)	18 (60)
≥5, No. (%)	12 (40)
Macroscopic portal vein invasion, No. (%)	
Yse	18 (60)
No	12 (40)
Extrahepatic spread, No. (%)	
Yes	16 (53.3)
No	14 (46.7)
BCLC stage, No. (%)	
B	6 (20)
C	24 (80)
Prior treatment, No. (%)	
Surgery	20 (66.7)
TACE/HAIC	22 (73.3)
MWA/RFA	21 (70)
Radiotherapy (including 125I implantation)	8 (26.7)
Anti-PD-(L)1 agent	14 (46.7)
Targeted therapy (except for lenvatinib)	10 (33.3)

*SD* standard deviation, *Kg* kilogram, *ECOG-PS* eastern cooperative oncology group-performance status, *WBC* white blood cells, *RBC* red blood cells, *PLT* platelets, *ALT* alanine aminotransferase, *AST* aspartate aminotransferase, *ALB* albumin, *TBil* total bilirubin, *PT* prothrombin time, *AFP* fetoprotein, *BCLC* barcelona clinic liver cancer, *TACE* transarterial chemoembolization, *HAIC* hepatic artery infusion chemotherapy, *MWA* microwave ablation, *RFA* radiofrequency ablation

### Safety

All patients who received the combination therapy were included in the safety analysis (Table 2). The most frequent all-grade treatment-emergent adverse events (TEAE) were fatigue (27 patients; 90%), followed by rash (25 patients; 83.3%), diarrhea (24 patients; 80%), and anorexia (12 patients; 40%). Most of them can be relieved after supportive therapy. Nine patients (30%) experienced grade 3 adverse events, including fatigue (13.3%), diarrhea (10%), anorexia (10%), hand-foot skin reaction (6.7%), and rash (3.3%). All the nine patients recovered to 1 or 2 AEs after dose reduction (Table 3). No grade 4 events and mortalities were observed.

**Table 2:** Incidence of treatment-emergent AE

AE	No. of patients (%)		
	All grades	Grade 3	Grade 4
Fatigue	27 (90)	4 (13.3)	0
Rash/desquamation	25 (83.3)	1 (3.3)	0
Diarrhea	24 (80)	3 (10)	0
Anorexia	12 (40)	3 (10)	0
Hypertension	11 (36.7)	0	0
Hand-foot skin reaction	9 (30)	2 (6.7)	0
Weight loss	9 (30)	0	0
Nausea	6 (20)	0	0
Fever	5 (16.7)	0	0
Mucositis, oral	5 (16.7)	0	0
Hemorrhage, nose	4 (13.3)	0	0
Cough	3(10)	0	0
Pain, abdomen NOS	2(6.7)	0	0
Vomiting	2(6.7)	0	0
Anemia	2 (6.7)	0	0
Leukopenia	2(6.7)	0	0
Thrombocytopenia	1(3.3)	0	0
Hyperbilirubinemia	5 (16.7)	1 (3.3)	0
ALT	4 (13.3)	0	0
AST	6 (20)	1 (3.3)	0
Hypoalbuminemia	4 (13.3)	0	0

*AE* adverse event, *NOS* not otherwise specified, *ALT* alanine aminotransferase, *AST* aspartate aminotransferase

**Table 3:** Dose delays and reductions due to adverse events

Patient ID	Any dose reduction (yes/no)		Reason of dose reductions	Mean daily dose, mg	
	LEN	GEF		LEN	GEF
001	yes	yes	fatigue, diarrhea	7.4	194.5
002	no	no	n/a	12	221.8
003	no	no	n/a	8	222.7
004	no	no	n/a	12	218.8
005	no	no	n/a	12	244.3
006	no	no	n/a	12	216.3
007	no	no	n/a	12	242.2
008	no	no	n/a	8	238.5
009	no	no	n/a	8	229.2
010	no	no	n/a	8	230.1
011	no	no	n/a	8	242.5
012	no	no	n/a	12	228.1
013	no	no	n/a	8	243.7
014	no	yes	rash, diarrhea	8	218.5
015	no	no	n/a	8	245.1
016	no	no	n/a	12	247.6
017	yes	yes	hand-foot skin reaction	7.5	206
018	no	yes	fatigue, anorexia	8	157.4
019	no	no	n/a	8	247.2
020	no	yes	fatigue, anorexia	12	222.4
021	yes	yes	diarrhea	11.1	168
022	no	yes	anorexia	12	205.5
023	no	no	n/a	8	241.9
024	no	no	n/a	8	238.3
025	no	no	n/a	8	245.6
026	no	no	n/a	12	236.1
027	no	yes	hand-foot skin reaction	12	207.2
028	no	no	n/a	8	221.8
029	no	no	n/a	12	221.8
030	no	yes	fatigue	8	230

*LEN* lenvatinib, *GEF* gefitinib

### Antitumor efficacy

**Fig. 2:**
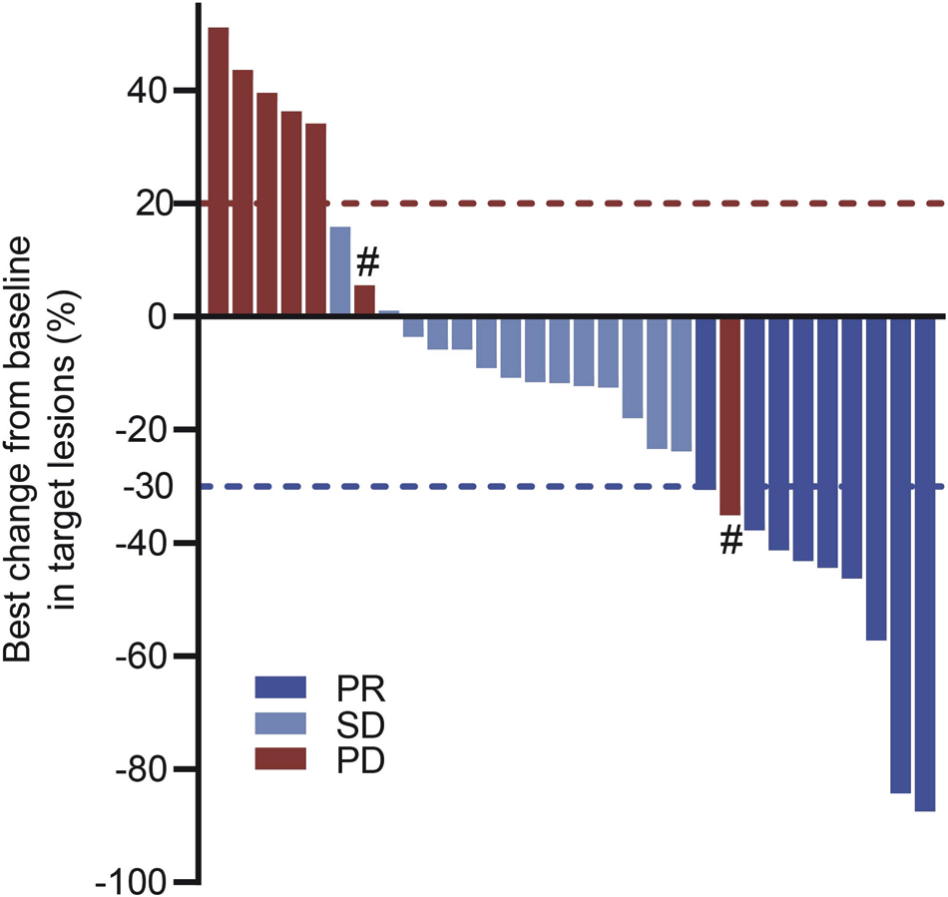
Best percentage change in sum of diameters of target lesions from baseline according to mRECIST. # indicating PD patients due to new measurable tumors. PR partial response, SD stable disease, PD progressive disease

**Fig. 3:**
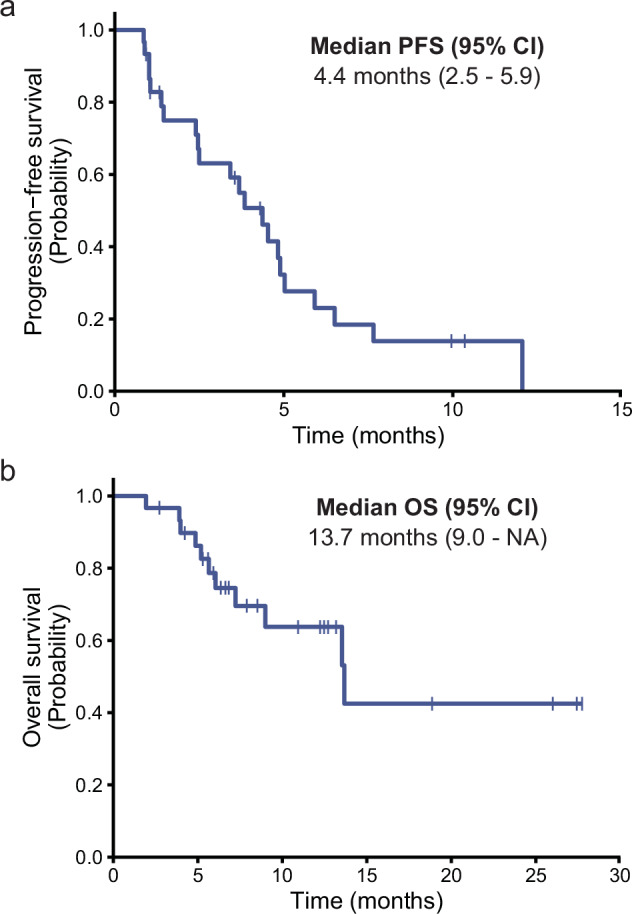
Kaplan–Meier curves of **a** progression free survival (PFS) and **b** overall survival (OS) for all 30 patients underwent combination treatment of lenvatinib plus gefitinib

In the efficacy analysis, 10 (33.3%) of 30 patients experienced tumor shrinkage by more than 30% from baseline in target lesions per mRECIST. One patient developed a new lesion, classifying this patient as having progressive disease despite regression of more than 30% of the target lesions (Fig. 2). Of the 30 patients treated with combination targeted therapy (lenvatinib plus gefitinib), 9 (30%) achieved a confirmed partial response (PR) and 14 (46.7%) had stable disease (SD) according to mRECIST criteria, with ORR of 30.0% and overall disease control rate (DCR) of 76.7%. Based on RECIST1.1, five (16.7%) achieved a confirmed PR and 18 (60%) had SD, with ORR of 16.7% and DCR of 76.7% (Table 4). One patient remained on study treatment for 12 months. One patient had confirmed PR according to mRECIST criteria at the first imaging assessment, however, this patient experienced treatment interruption owing to deteriorating liver function. Another confirmed PR patient (based on mRECIST criteria) experienced treatment interruption due to hemoptysis and then withdrew from the study owing to subsequent ruptured intracranial aneurysms. After discontinuation of the study, imaging evaluation confirmed disease progression. Three patients had confirmed SD according to mRECIST criteria at the first imaging assessment, however, they withdrew consent and received other therapies. The estimated median PFS and OS time was 4.4 months (95% CI: 2.5 to 5.9) (Fig. 3a) and 13.7 months (95% CI: 9.0 to NA) (Fig. 3b), respectively. Three patients continued on study treatment at the time of this report.

**Table 4:** Summary of best response

Best response	No. of patients (%)	
	RECIST 1.1	mRECIST
CR	0 (0)	0 (0)
PR	5 (16.7)	9 (30)
SD	18 (60)	14 (46.7)
PD	7 (23.3)	7 (23.3)
ORR	5 (16.7)	9 (30)
DCR	23 (76.7)	23 (76.7%)

*CR* complete response, *PR* partial response, *SD* stable disease, *PD* progression disease, *ORR* objective response rate, *DCR* disease control rate

## Discussion

Liver cancer is still the most common type of malignant tumor in the world, but clinical drugs are still far from meeting the treatment needs of HCC patients. As the first FDA-approved drugs for advanced HCC patients, sorafenib only provides about 3 months longer OS than placebo.^18^ After more than 10 years, the REFLECT trial showed the non-inferiority of lenvatinib over sorafenib in OS, and lenvatinib achieved improved ORR as compared to sorafenib (24.1% vs 9.2%),^9^ resulting in FDA approval of lenvatinib for first-line treatment of patients with unresectable HCC in 2018. Recently, based on the results of phase III clinical trial of IMbrave 150, the FDA approved the use of altezumab and bevacizumab for advanced HCC. In this trial, the combined therapy showed 33% ORR.^19^ Current approved second-line therapies for HCC are cabozantenib (ORR 4%),^20^ regorafenib (ORR 11%),^21^ ramucirumab (ORR 5%),^22^ and pembrolizumab (ORR 18.3%).^23^ Compared to these established second-line therapies, our results showing that lenvatinib plus gefitinib achieved ORR of 16.7% based RECIST1.1 and 30% based mRECIST in HCC patients progressed after lenvatinib treatment compare favorably. It should be noted that 60% of the patients in this study had received second or third-line systemic therapy before enrollment. A large-scale clinical trial will be designed and initiated to further validate the therapeutic efficacy of this combination in the near future. In addition, for patients with recurrence after liver transplantation or patients who can not receive immunotherapy for other reasons, new treatment schemes still need further exploration. Our dual targeted therapy will not be limited by this, and its curative effect is worth expectation in above subset HCC patients.

Because of the extensive clinical application of EGFR inhibitors in cancer patients, their toxicity and pharmacokinetics are well known. In previous clinical trials, erlotinib, an EGFR inhibitor, showed good tolerance, but its therapeutic efficiency in HCC patients was limited.^24,25^ Additionally, a phase III SEARCH trial showed that erlotinib plus sorafenib as a first-line treatment in advanced HCC patients did not result in significant clinical benefits to HCC patients.^26^ These results indicate that the currently observed synergy with EGFR inhibition is specific to lenvatinib, as also seen in vitro.^11^ Unlike sorafenib, lenvatinib can specifically inhibit FGFR family members. Our previous study demonstrated that lenvatinib synergizes with EGFR inhibitors mainly by abrogating the MAPK signaling mediated by FGFRs and EGFR, respectively, which may explain the main reasons for the failure of the above phase III SEARCH trial.^11,26^ Subsequently, a number of related studies confirmed that combination of EGFR and FGFRs inhibition can achieve promising antitumor effect in various cancer types, such as FGFR-driven urothelial cancer,^27^ FGFR2 fusion-positive cholangiocarcinoma,^28^ and non-small cell lung cancer (NSCLC).^29^

Our trial is a first-in-human, proof-of-concept, combination treatment study of EGFR inhibitor and lenvatinib in HCC patients. This combination not only achieved impressive anti-tumor efficacy, but also showed acceptable AEs in most HCC patients, with only one patient withdrawing due to a grade 3 AE. Moreover, these symptoms can improve quickly after dose reduction or a short period of drug withdrawal. It is worth noting that another phase I clinical trial is underway to evaluate the clinical efficacy of EGFR monoclonal antibody, cetuximab and levatinib in the treatment of patients with head and neck squamous cell carcinoma (HNSCC). According to the report of the meeting of American Society of Clinical Oncology (ASCO) in 2020, among 9 patients with HNSCC who received cetuximab plus levatinib, 6 patients had a PR with 67% ORR.^30^ These results indicate that a combination of lenvatinib and EGFR inhibitor may have clinical benefits for different types of human cancers.

Rational drug combinations can help avoid both intrinsic and acquired resistance to targeted agents.^31^ Unfortunately, drug resistance is still inevitable in the scenario of combination treatment. Despite response rates have been increased by combining lenvatinib with EGFR inhibitor, the majority of HCC tumors still fail to regress and durability of disease control remains a challenge. Although EGFR-mediated signaling pathway is one of the major ways of drug resistance to lenvatinib, there are still other EGFR-independent drug resistance mechanisms, which can confer drug resistance to lenvatinib monotherapy as well as lenvatinib plus EGFR inhibitor combination therapy. It was reported that acylphosphatase 1 (ACYP1) had a direct regulatory role in glycolysis and drove lenvatinib resistance of HCC via the ACYP1/HSP90/MYC/LDHA axis.^32^ Another study demonstrated that hypoxia-responsive PPARGC1A/BAMBI/ACSL5 axis promoted progression and resistance to lenvatinib in HCC.^33^ Moreover, cancer-associated fibroblast-derived secreted phosphoprotein 1 (SPP1) also enhanced lenvatinib resistance in HCC via bypass activation of RAF/MAPK and PI3K/AKT/mTOR, and promoted epithelial-to-mesenchymal transition (EMT).^34^ In our current study, 7 out of 30 EGFR^high^ HCC patients showed direct and rapid disease progression during the combination treatment of lenvatinib plus gefitinib. It is possible that the above-mentioned resistance mechanisms are present in these PD patients. In spite of such resistance mechanisms, the present data warrant a further investigation into the combination of lenvatinib and EGFR inhibition in HCC.

There are several limitations in the present study. Due to ethical issues, expression of EGFR was analyzed in the tumor samples from the patients taken at first surgery or puncture after diagnosis. It is unclear whether EGFR expression changes during the evolution of HCC after first diagnosis. Thus, a biopsy taken just prior to the start of the combination therapy may provide a more accurate assessment of EGFR expression in these patients. Peripheral circulating tumor DNA (ctDNA) profile provides a non-invasive method to capture genetic heterogeneity across cancer lesions and identify cancer-specific mutations. In a national cohort study of 1616 HCC patients who underwent ctDNA analysis, the incidence of EGFR amplification was 6.5%, which may be related to the drug resistance to lenvatinib.^35^ Therefore, the detection of *EGFR* alterations in ctDNA can potentially be used to assist in screening subset patients who may benefit from treatment of lenvatinib plus gefitinib. In addition, most patients had undergone multiple local and systemic therapies prior to enrollment, which may affect the effectiveness of combination therapy. Well-desigend, preferably randomized, phase II trial is therefore needed to further validate the efficacy of this combination therapy. Third, the enrollment and follow-up were influenced by COVID-19.

In conclusion, the combination therapy showed an initial therapeutic effect and was well tolerated, worthy of further clinical study.

## Materials and methods

### Study design and participants

This was a single-arm, open-label, exploratory study, which aimed to investigate the use of lenvatinib plus gefitinib in treating patients suffering from HCC who had either primary or acquired resistance to lenvatinib. Primary resistance was defined as tumor progression observed at the first follow up and acquired resistance was defined as complete response, partial response, or stable disease determined at the first review and then tumor progressed at the subsequent follow up. The study was carried out at Renji Hospital in China from December 2020 to March 2023. Expression of EGFR was analyzed in the tumor samples of the patients according to our previous study.^11^ In order to assess the safety and tolerability of the combination therapy (lenvatinib plus gefitinib) in patients, gefitinib (also called by its trade name Iressa; Astra Zeneca) was initially administered at half of the normal clinical dose, that is, 125 mg per day. If the patient could tolerate well after 1 week, then the dose was adjusted to 250 mg per day. Lenvatinib (also called by its trade name Lenvima; Eisai) was administered at the normal clinical dose (body weight <60 kg, 8 mg per day; body weight ≥60 kg, 12 mg per day). For patients who experienced grade 1 or 2 toxicities, supportive therapy was permitted before consideration of dose reduction. For patients who had grade 3 toxicities, study drugs were decreased alternately for each occurrence of this adverse event (AE). This reduction was carried out in a step-by-step manner, beginning with gefitinib, and continued until the toxicity resolved to grade 2. Treatment was discontinued for patients who had any grade 4 toxicity. The treatment went on until one of the following situation occurred: disease progression, development of unacceptable toxicity, or withdrawal of consent.

Inclusion criteria included: (1) age 18 to 75 years; (2) stage B or C categorization based on the Barcelona Clinic Liver Cancer (BCLC) staging system; (3) at least 1 measurable target lesion in liver; (4) progression after standard treatment; (5) no response or resistance to lenvatinib; (6) Child-Pugh class A (score, 5–6) or B (score, 7); (7) platelet count ≥60 × 10^9^/L, PT time extension ≤6 s; (8) Eastern Cooperative Oncology Group performance status (ECOG PS) of 0 or 1. Exclusion criteria included: (1) uncorrectable coagulation disorders, accompanied by overt bleeding tendency; (2) patients are required to undergo long-term anticoagulant or antiplatelet treatment; (3) patients suffering from an unstable/active ulcer or gastrointestinal bleeding; (4) heart disease in need of treatment or high blood pressure which is not well managed; (5) patients suffering from interstitial pneumonia; (6) hepatic encephalopathy or intractable ascites; (7) There is a definite active infection; (8) patients had received radiotherapy, chemotherapy and/or interventional therapy within 4 weeks prior to the commencement of the study; (9) severe dysfunction of vital organs, for example, serious cardiopulmonary dysfunction; (10) other concomitant anti-tumor therapies; (11) the investigator judged that the patient was incapable or reluctant to follow the protocol.

The study was registered at ClinicalTrials.gov (NCT04642547). The trial protocol was approved by the institutional review board of the Renji Hospital, Shanghai Jiaotong University School of Medicine (KY2020-159). Written informed consent was provided by all patients before inclusion.

### Follow-up, assessments and outcomes

The primary end point was ORR, defined as the ratio of patients achieving complete and partial response to the total patient population. A secondary end point was PFS, defined as time interval from the start of combination therapy (lenvatinib plus gefitinib) to first tumor progression or death due to any reason. The other secondary end points encompassed DCR and OS. DCR was defined as the proportion of patients whose best response was a complete response, partial response, or stable disease lasting for ≥28 days. OS was defined as the time from the start of combination therapy (lenvatinib plus gefitinib) to death due to any cause, and patients remaining alive were censored at the date of last contact. The overall response was assessed by means of RECIST1.1 and mRECIST, respectively. Patient evaluations were carried out prior to the combination therapy. Subsequently, they were conducted every 4 weeks until week 12, and then at intervals of 8–12 weeks. At each visit, all AEs were documented and evaluated in accordance with the National Cancer Institute Common Terminology Criteria for Adverse Events version 3.0. Meanwhile, enhanced CT/MRI scans and laboratory evaluations, including complete blood count, chemistry, coagulation, and AFP were finished. Followe-ups were conducted for patients until death or the end of the study (March 31, 2023).

### Statistical analyses

The statistical analyses were carried out by means of the SPSS 22.0 statistical software (SPSS, Chicago, IL) and Graphpad Prism5 (Graphpad software Inc.). The patient characteristics were summarized using median along with the standard deviation (SD) for continuous variables, and using percentages for categorical variables. PFS and OS were analyzed using the Kaplan–Meier method.

## Supplementary information

Trial Protocol

## Data Availability

Further information and resources are available from the corresponding authors upon request. Inquiries should be directed to and will be fulfilled by Bo Zhai (zhaiboshi@sina.com).
