# Supplementary material for: Efficacy and safety of lenvatinib plus gefitinib in lenvatinib-resistant hepatocellular carcinomas: a prospective, single-arm exploratory trial
Source: Signal Transduct Target Ther. 2024 Dec 9;9:359. doi: 10.1038/s41392-024-02085-8 (PMC11628597; doi:10.1038/s41392-024-02085-8)
Supplement: Supplementary file 1 — Trial Protocol [file 41392_2024_2085_MOESM1_ESM.docx]

**A Prospective Clinical Study of Lenvatinib Combined With Gefitinib in the Treatment of Lenvatinib-resistant Hepatocellular Carcinoma**

**Background:**

Lenvatinib is the only targeted drug approved by the FDA for the first-line treatment of advanced liver cancer after sorafenib. However, clinical trial data showed that the survival benefit of lenvatinib for liver cancer patients is very limited, and the response rate is less than 20%, suggesting that a large number of liver cancer patients do not respond to lenvatinib treatment.

Our latest research found that knocking out EGFR can increase the sensitivity of liver cancer cells to lenvatinib. Subsequent verification experiments with EGFR inhibitors (erlotinib or gefitinib) and lenvatinib found that the combination therapy has a significant synergistic therapeutic effect on most liver cancer cell lines with high level of EGFR. The synergy is mainly based on the synergistic inhibition of ERK/MAPK signaling pathway by lenvatinib and EGFR inhibitors. Therefore, the combined use of EGFR inhibitors and lenvatinib is expected to significantly increase the response rate and survival of clinical liver cancer patients. Especially for patients with advanced liver cancer who do not respond to lenvatinib monotherapy at the initial stage, this treatment strategy will be a new option.

**Objectives of Study：**

To observe and determine the safety and efficacy of Lenvatinib combined with Gefitinib in the treatment of Lenvatinib resistant hepatocelullar carcinoma.

**Study design:**

This study is a single-arm, single-center, open-label experimental study

**Research object:**

Patients with advanced liver cancers who are unresponsive or resistant to lenvatinib treatment

**Sample size：**30

**Diagnostic criteria:**

Meets AASLD or EASL clinical liver cancer diagnostic criteria

**Inclusion criteria:**

Inclusion criteria: (1) Unlimited gender, aged 18-75 years; (2) Meets American Association for the Study of Liver Diseases (AASLD) or European Association for the Study of the Liver (EASL) clinical diagnostic criteria of hepatocellular carcinoma; (3) Barcelona Clinic Liver Cancer (BCLC) Stage B or C, and there is at least one measurable tumor in the liver (long diameter ≥ 1cm);(4) progressing after standard treatment; (5) Unresponsive or resistant to Lenvatinib; (6) Child-Pugh A or scored 7 B; (7) Eastern Cooperative Oncology Group performance status score <= 1; (8) Platelet count >= 60x10^9/L, Prothrombin time prolonged <= 6 seconds.

**Exclusion criteria：**

Exclusion criteria：(1) Uncorrectable coagulopathy with obvious bleeding tendency; (2) Patients need long-term anticoagulant or antiplatelet therapy and cannot stop the drugs; (3) Patients with unstable or active ulcer or gastrointestinal bleeding; (4) Heart disease requiring treatment or not well controlled high blood pressure; (5) Patients with interstitial pneumonia; (6) Hepatic encephalopathy or refractory ascites requiring treatment; (7) There is a clear active infection; (8) Receiving radiotherapy/chemotherapy/interventional therapy for tumor within 4 weeks before the start of the study; (9) Severe insufficiency of important organs, such as severe cardiopulmonary insufficiency; (10) Other accompanying anti-tumor treatments; (11) The investigator assessed that the patient was unable or unwilling to comply with the protocol.

**Baseline examination:**

- Laboratory evaluation：blood routine, liver and kidney function, electrolytes，coagulation, tumor markers such as AFP, EGFR expression in tumor biopsy.
- Imaging evaluation: liver enhanced MRI/CT, chest CT scan.

**Treatment:**

- Dose selection/adjustment: Gefitinib is taken 125mg/day orally, and lenvatinib is taken orally at the clinically normal dose (weight ≤60Kg, 8mg/day; weight>60Kg, 12mg/day). The dose of gefitinib will be adjusted to 250mg/day if the patient is well tolerated one week after the combined medication. The Lenvatinib dose will remain unchanged.
- Standards for combination medication: Patients can not accept other local and systemic anti-tumor therapies during the study, including hepatic artery chemoembolization, ablation therapy, radiotherapy (including implantation of interstitial seeds), surgical resection, and systemic chemotherapy.
- Remedial medicine and supportive treatment: Patients with intolerable adverse reactions must stop the combination medication and give corresponding symptomatic supportive treatment.

**Early withdrawal/termination criteria:**

(1) Treatment will be terminated when disease progression was confirmed unless the

following 3 items are met:

- Observed disease progression is judged based on a single new lesion that is not considered clinically significant
- The researcher believes that the symptom or performance score has not changed compared with pre-combination therapy
- The investigator believes that it is not necessary to give other treatments immediately.

(2) The investigator judge that it is inappropriate to continue the trial when patients encounter intolerable adverse event or deterioration of disease

(3) Patients accept other not allowed anti-tumor treatment during the study, including but not limited to radiotherapy, chemotherapy, surgical treatment, etc.

(4) Patients have the right to choose to continue or withdraw from the study at any stage of the study

(5) The researcher judges that patients could not afford to the trial due to their serious condition

(6) Intolerable toxicity occurs

**Follow up:**

Patients will undergo follow-up review every month (±7 days) for the first 3 months, and every 2-3 months thereafter.

- Blood routine
- Liver and kidney function, electrolytes
- Coagulation
- Tumor markers such as AFP
- Imaging evaluation of tumor response to therapy
- Medication
- Adverse events

**Treatment after progression:**

When tumor progression is confirmed during the follow up, patients should be re-evaluated and given the standard treatment according to BCLC stage. However, follow-up will be encouraged until the end of the study or death.

**Study endpoint:**

Primary endpoint: Objective response rate (ORR)

Secondary endpoint: Progression free survival (PFS), Overall survival (OS) and Disease control rate (DCR)

Safe endpoint: The proportion of adverse events after treatment for each patient is statistically described.

**Efficacy evaluation criteria:**

The efficacy evaluation refers to the modified solid tumor efficacy evaluation standard (mRECIST)

| CR | Disappearance of any intratumoral arterial enhancement during in target lesions |
| --- | --- |
| PR | ≥ 30% of the sum of the diameters of viable portions (enhancement on arterial phase) of target lesions taking as reference the baseline sum |
| PD | ≥ 20 % of the sum of the diameters of viable (enhancing) portions of target lesions taking as reference the smallest sum of the diameters of viable portions of target lesions recorded since the start of treatment |
| SD | Neither response nor progression |

**Statistical Analysis Plan:**

SAS (version 9.2 or higher) will be used for statistical analysis. Continuous variables will be analyzed using descriptive statistical methods (such as number of cases, mean, median, standard deviation [SD], minimum and maximum). Categorical variables are analyzed using frequency tables (frequency and percentage).
